# Parent Experiences of Child Loss and End-of-Life Care in a Pediatric Intensive Care Unit: Protocol for a Qualitative Study

**DOI:** 10.2196/43756

**Published:** 2023-03-22

**Authors:** Sara Alcón Nájera, Maria Teresa González-Gil

**Affiliations:** 1 Gregorio Marañon Hospital Madrid Spain; 2 Autonomous University of Madrid Madrid Spain

**Keywords:** hospice and palliative care nursing, intensive care units, pediatric, patient-centered care, family nursing, death, qualitative research

## Abstract

**Background:**

Death of a child in the pediatric intensive care unit is a rare event that can occur after failed cardiopulmonary resuscitation efforts, after a brain death diagnosis, or after a decision to limit therapeutic efforts. Nevertheless, even in the case of children with terminal and progressive illnesses, death is a crisis that comes as a surprise to parents and is perceived as unexpected. In the final stage of a child’s life, health care staff play a key role in sharing feelings and experiences with the family and in supporting them throughout the process in order to facilitate the grieving process.

**Objective:**

The aim of this study is to explore the experiences of parents whose children have died in a pediatric intensive care unit.

**Methods:**

To address the study aims, a qualitative phenomenological study based on the van Manen proposal will be carried out. The study will be conducted in the pediatric intensive care unit of a tertiary care hospital. The study population will be parents or guardians (older than 18 years) of children who have died in the unit at least 6 months prior to potential participation in the study. Purposive sampling will be used to ensure sample diversity in relation to experiential variables. Families will be initially contacted by letter sent alongside the standard letter of condolences from the hospital, and then recruited in a subsequent telephone call. The sample size will be determined by data saturation. In-depth interviews will be conducted individually or in pairs. Parents will decide when, how, and where to conduct the interviews, which will be transcribed verbatim and examined using thematic discourse analysis.

**Results:**

This study was awarded a grant in December 2020 and was approved by the Medical and Health Research Ethics Committee on December 21, 2020. Data collection started in April 2021, and the results are expected to be published in 2023.

**Conclusions:**

This project is intended to maintain, strengthen, and build on a particular line of research on end-of-life care with a focus on effective coping, spiritual well-being, and the adaptive grieving process. The results will contribute to establishing action guidelines that are both based on the discourses of parents who have experienced the death of a child and geared toward high-quality end-of-life care through dignified death and adaptive grief management.

**International Registered Report Identifier (IRRID):**

DERR1-10.2196/43756

## Introduction

Child and adolescent death caused by noncurable diseases is still a present reality despite developments in pediatric medicine [1]. According to the World Health Organization data [2], 6,073,010 children under the age of 14 years died in 2019 around the world, leaving an intense, lasting impact on their families.

Death produces anxiety, distress, depression, and fear, especially when the deceased is a child. As children exhibit different characteristics from adults, the approach to palliative and end-of-life care for these patients and their families should also be different. The primary objective is to ease suffering through proper symptom control, psychological, social, and spiritual support, and preparation for death and grieving. The maladjustment process that occurs within the family is particularly relevant, as it complicates the acceptance of their loss. The sorrow arising from this loss, coupled with the ongoing decline in the structure and function of the family unit, makes it difficult to develop coping strategies and worsens the experience of grief. Even in the case of children with terminal and progressive illnesses who experience life-threatening events, death is a crisis that comes as a surprise to parents and is perceived as unexpected [3].

In pediatric end-of-life care [4], it is widely accepted that the family's experiences in the hospital can have a significant long-term impact on memories and the grieving process [5]. There is therefore an important incentive to improve the quality of care provided in these circumstances, both to improve patient experience as well as to support the impending grief of families. Meert et al [6] identified a high incidence of complicated grief symptoms in parents whose children had died in the pediatric intensive care unit (PICU). In their study, they note that these symptoms lessen between 6 and 18 months after the child’s death, although not for all parents. In light of the above, we believe it is relevant to investigate the experiences of the families of children who have died in the PICU to better discern the actions of health care workers; how to provide the family with the most appropriate resources needed to ease their grieving process; how to foster a supportive atmosphere and a dignified death; and to assess whether the care provided by health care workers is satisfactory.

Over the last decade, pediatric care has seen improvements in the care of critically ill children, most notably, the adoption of the family-centered care (FCC) model. This model is based on the premise that parents are the people who know their children best [7] and should therefore be included in the caring relationship, as their involvement improves patient outcomes. The definition of FCC encompasses 4 fundamental concepts: respect and dignity; information sharing; participation in care and decision-making; and collaboration between patients, families, and the health care team. In addition to the physical benefits for patients, when parents are involved in their children’s daily care by playing an active role in the care team, emotional and psychological aspects improve through effective coping.

Death of a child in the PICU is a rare event that can occur after failed cardiopulmonary resuscitation (CPR) efforts, after a brain death diagnosis, or after a decision to limit therapeutic efforts (LTE). According to data provided by the MOMUCIP study [8], which explores the causes of death in PICUs, the most frequent cause of death was after a decision to LTE (n=151, 50.7% of all cases, widely ranging between 19.2% and 64.6% depending on the hospital), followed by indicated but failed CPR (n=114, 33.8%), and brain death (n=52, 15.4%). The main reason for LTE was failure to provide CPR for cardiac arrest (n=45%), followed by withdrawal of mechanical ventilation (n=48, 31.5%), and administration of vasoactive drugs (n=33, 21.6%).

Technological advances have increased the ability to provide support in very critical situations. However, life does eventually come to a natural end, and the continuation of therapeutic interventions is not always suitable, sometimes raising the question of whether continuing treatment is in the child’s best interest. If the decision is made to withdraw life-support therapy, the main focus then shifts from an interventionist approach (aimed at sustaining life) to an approach aimed more at providing as much comfort and emotional support as possible to the child and their family. In the final stage of a child's life in PICUs, health care staff play a key role in sharing feelings and experiences with the family and in supporting them throughout the process. Providing high-quality end-of-life care for patients and their families eases suffering through appropriate decision-making and symptom management, which helps to facilitate the grieving process.

In relation to the available evidence, Mitchell and Dale [9] highlight the importance of providing anticipatory care for patients with life-limiting illnesses by planning care in advance together with the family to let them make decisions based on clear information and to avoid unnecessary interventions. Regarding the planning and implementation of palliative care in intensive care units, it is neonatal units that have made the most progress in this area and where the vast majority of scientific evidence is available. As early as 2002, Catlin and Carter [10] established the key elements necessary for an effective approach to palliative care, forming the basis for numerous subsequent studies and protocols, serving as an example of a high-quality practical guide [11].

Recent studies address a range of issues, including evaluating the implementation of a care program in neonatal intensive care units (NICUs) [12]; the benefits of implementing interventions that focus on early symptom management; the possibility of planning interdisciplinary meetings to discuss issues such as end-of-life care (ie, anticipating complications and treatment options or not starting treatment or resuscitative measures); and the promotion of joint decision-making for neonates with severe and incurable illnesses (ie, morbidity and mortality associated with the condition). These studies also emphasize the need to train nurses in palliative and end-of-life care, as lack of training is one of the barriers that may be encountered [13] when preempting symptom recognition in the child and their family and when attempting to improve nursing care and support. The literature also stresses the need to continue new lines of research involving health care workers who care for children with conditions requiring palliative and end-of-life care and for their families [14]. The creation of clinical practice guidelines where palliative and curative approaches coexist and where the timing of their transition is established is also of vital importance, as there is ample evidence of the benefits of palliative care in PICUs and NICUs. However, the number of publications successfully reporting such benefits through protocols and care programs is scarce [11].

All of the above brings to the forefront the fundamental roles that nurses play in end-of-life care [15]. This is further accentuated in intensive care environments and even more so in the pediatrics branch, in the case of FCC. The role of the nursing professional takes on greater importance when it comes to supporting the patient and their family at the end of the patient’s life. This is due to the therapeutic relationship based on trust and support that is established between them, which improves the family’s ability to cope. Similarly, several nursing theories and models throughout history have considered palliative care as a significant part of nursing functions [16], creating a framework for action of its own. According to Wu and Volker, the Humanistic Nursing Theory approaches the field of palliative care through the lens of the bond created between the nurse and the patient, seeking to provide patient comfort, encourage decision-making, and maintain dignity [15].

There is also a major pillar of nursing that illustrates the importance of nurses in end-of-life care—the nursing care process (NCP). This, together with the North American Nursing Diagnosis Association taxonomy, allows specific nursing diagnoses directly related to palliative care to be identified, for example, risk for maladaptive grieving (00302), maladaptive grieving (00301), and readiness for enhanced grieving (00285) [17,18]. Through the NCP, and based on standardized language, nurses carry out interventions specific to their discipline (Nursing Interventions Classification) such as active listening, anxiety reduction, coping enhancement, and dying care, which give them a sufficient degree of autonomy when it comes to achieving measurable outcomes (Nursing Outcomes Classification) [19], which in turn contribute to the resolution of patient problems.

For these reasons, the general aim of this study was to explore the experiences of parents of children who have died in the PICU. In addition, the specific aims of this study were the following: to explore parents’ perceptions of their capacity for self-determination and active participation in the process of caring for their child at the end of their life; to identify needs perceived by parents when saying goodbye to their deceased child; and to identify limitations regarding family support as perceived by parents and strategies for improvement.

## Methods

### Design

The project will be based on the constructivist paradigm, and a qualitative phenomenological study will be carried out according to the van Manen methodological proposal. According to this author, phenomenological research is the study of life experiences, of the lifeworld and everyday life (which constitutes the prereflective experience), the nonconceptualized or noncategorized experience [20]. In keeping with the above, an interpretative phenomenological or hermeneutic approach will be taken, as it provides an understanding that there is no single reality but multiple realities [21], which depend on the subjective interpretations of the individuals who experience a given phenomenon, the life stage that they are at when that phenomenon occurs, the place they are in, and their interactions with the people around them) [22].

We propose an intersubjective approach to the study phenomenon, which means seeking to gain access to the multiple interpretations of reality through the subjectivities of the families who have experienced first-hand the loss of a child in the PICU while at the same time recognizing the active participation of researchers themselves in the research process [21,22].

### Study Setting

The study will be conducted in the PICU at the Gregorio Marañón General University Hospital in Madrid, Spain. This is a multipurpose unit that provides clinical care for critically ill children and coordinates and supports other hospital services in the prevention and treatment of life-threatening situations, as well as in the care of complex technology–dependent patients. It has an intensive care and an intermediate care hospitalization area with a total of 17 beds and an occupancy rate of 70%-80%.

The reasons for admission of PICU users, according to their clinical records, were as follows: heart conditions or postoperative cardiac surgery (23%); respiratory (30.4%), hemodynamic (14.2%), hemato-oncological (3.5%), neurological, gastrointestinal (2.9%), or renal problems (2.7%); multitrauma (5.9%); and infection or acute sepsis (6.5%), transplant (3.9%), and so forth. During the last 3 years (2018-2020), an average of 385 children were admitted to the PICU per year, with a mortality rate of 4.6%, 1.9%, and 2.9%, respectively. The most frequent causes of death were limitation of therapeutic efforts, multiorgan failure, and brain death [23].

### Participants

The study population will include parents or guardians, older than 18 years, of children who have died in the PICU at least 6 months prior to their potential participation in the study [9], who are able to communicate verbally or in writing, and who voluntarily agree to participate in the study. Participants will be assured that their participation and the subsequent interview will not put them at any emotional or psychological risk.

In relation to the grieving process, the literature consulted recommends different time periods for inviting families to participate, ranging from 4 [24] to 12 months [25] after the death of their child. However, each family’s circumstances will be assessed during the phone interview, ensuring that all stages of grief have been overcome successfully, and avoiding significant dates such as birthdays and anniversaries.

All persons whose participation in the study may put them at risk of emotional instability, such as parents who are going through pathological or complicated grief, will be excluded [26].

Purposive sampling [27] will be used to ensure sample diversity regarding experiential variables. These variables will be determined by the research team’s consultation of available evidence and prior experiential knowledge (Multimedia Appendix 1) and attempts will be made to ensure access to varied, meaningful, and competing narratives. In more advanced stages of the research process, a decision will be made to follow either a theoretical sampling method that includes new variables or experiential criteria, which emerge from the analysis.

For the recruitment of families, together with the institutional letter of condolences sent by the unit, they will be asked in writing about the possibility of participating in the study. In a later telephone call, the project will be explained to them in greater depth, any doubts or questions will be addressed and compliance with the inclusion criteria will be assessed.

The final sample size will be driven by data saturation, that is, the occurrence of redundant and repeated information [27]. This stage of the research will end when a solid set of primary protocol materials (interviews, observations, and notes) has been collected and described and there are enough data to perform a comprehensive categorization or classification which, in turn, can inform sound analysis, interpretation, and theorization, leading to valuable results [28].

However, after consulting the available literature, it is estimated, in terms of time and budget planning, that data saturation can be achieved after a total of between 10 [9,25] and 25 parent interviews. These sample sizes are taken as a reference, but results will depend on the interviewer’s expertise, the richness of participant discourse, and the analyst’s ability to see beyond the data (or the analyst’s theoretical sensitivity), among other aspects [29].

### Data Collection

In-depth individual or paired interviews will be conducted according to each family’s preferences (paired interviews allow family practices to be analyzed and shared family realities to be co-constructed) [30]. The interview is a technical instrument that is very much in tune with theoretical hermeneutic phenomenology, as it is intended to explore and collect experiential narrative material and to be the vehicle for investigating the meaning of that experience. Given the sensitive nature of the topic, a script for the semistructured interviews will help the interviewer to focus on the most relevant aspects of the experience while ensuring that emotional vulnerability is carefully managed [20]. Although the interview script is not meant to be a closed framework, it does allow for some control over the most emotionally sensitive areas and the routes to reach them if the participant is not prepared.

Based on the general and specific study objectives and taking the researchers’ experiences into consideration, carefully worded dynamic interview questions have been drafted in order of the most general to the most specific, and from the least sensitive to the most sensitive (Multimedia Appendix 2). The script will be flexible in nature and will evolve according to the fieldwork and the needs arising from it. The information gathered and topics emerging from this information will refocus the approach and the collection of additional new data [28].

The families who agree to participate will decide when, how, and where they want to be interviewed: in person, with different locations available (travel expenses will be covered), or remotely, by videoconference or telephone. These will be audio-recorded. The recordings will be kept for up to 1 year after the publication of the study results in case further analysis is required. They will then be deleted.

While the interviews are being conducted, through close observation [29], the data obtained from nonverbal communication will be recorded in a field notebook. This is how, according to van Manen [32], we come as close as possible to the participant's lifeworld, so that we can grasp the meaning of that person's particular experience. This register can help to contextualize the families’ discourses and to understand how the death of a child in the PICU impacts their parents in greater depth. Participant files will also be created where sociodemographic, clinical (reason for admission, time spent in the PICU, date, and cause of death), and relational data will be collected to help understand and contextualize their discourses. Some data will be retrievable from their clinical records, while others will have to be completed during the interviews.

### Data Analysis

The van Manen hermeneutic approach [32] will be used for the qualitative analysis of the data. The interviews will be transcribed verbatim, and a thematic discourse analysis will be performed by identifying the themes that give meaning to the text and the experience. The themes are interpreted as the significance of the experience itself—attributing meaning and significance to the phenomenon at hand in an attempt to understand it, in such a way that each theme describes a different facet of the experience being faced. As themes emerge, an attempt will be made to capture the thematic status networks in more phenomenologically sensitive paragraphs by making reflective-theoretical notes (memoing) that will result in a creative hermeneutic process [20].

Subsequently, the themes that will inform the phenomenological description will be established: since not all the meanings that we find when reflecting on a phenomenon or lived experience are unique to that phenomenon or experience, this means that the key themes will be identified. To this end, we will identify the main qualities of each of the themes that are quintessential to that phenomenon.

For coding, the support software ATLAS-ti (Estudiantes; L-BFB-FEC, individual license) will be used for structuring the analysis and storage of data. This program will facilitate the identification of codes, the creation of the first themes for analysis, and the initial exploration of all written documents arising from the interviews for the study of qualitative data [33].

During the analysis process, the results will be cross-checked and compared with those of similar studies to verify how they emerge from different perspectives or broader theoretical frameworks and to better explain what the study really means. Comparing and contrasting emerging theoretical proposals with those of other researchers will lead to greater integration and enrichment of the study area's body of knowledge [28]. This comparison will lead to the reformulation, restructuring, extension, or refinement of previous theoretical constructs. The constant comparative method is a way of generating theory from the comparative and systematic analysis of data [34]. In order to synthesize the final findings, efforts will be made to integrate all the themes into a coherent and logical whole, with evocative meaning.

### Ethical Considerations

Parents’ principle of autonomy [35] will be preserved by signing the informed consent form after reading an information sheet that will be provided to them, containing the rationale for the project, a brief explanation of the study, who is involved, and the risks and benefits associated with their participation. Permission will be requested from participants to be audio-recorded. All participant questions will be answered before signing the informed consent form. They will choose to participate without any pressure or coercion. Informed consent will be written using the informed consent form designed for this purpose and approved by both ethics committees.

All interviews will be attributed codes, eliminating any personal details of the informants. Confidentiality will be preserved throughout the study, and the safety of participants will be ensured at all times, bearing in mind that the study is sensitive in nature [36]. The project was approved by the Clinical Research Ethics Committee at the Gregorio Marañón Hospital (CuidFin_Enf1; January 12, 2021), as well as by the Research Ethics Committee at the Autonomous University of Madrid, Spain (CEI-121-2451; February 11, 2022).

### Rigor

To ensure the internal validity of the project (credibility and accuracy), the following will be used: several forms of triangulation [22] of data collection techniques from interviewers and analysts; audit strategies, for example, member checking [37] or feedback from participants and the “critical friend” strategy [38] or feedback from critical experts); and thorough descriptions of the research process and methodological decision-making.

With regard to external validity (transferability), a detailed description of the characteristics of the participants and the study setting will be provided in order to contextualize the findings and assess the relevance of the gathered evidence for application in other clinical settings.

In addition, auditability will be ensured so that other researchers wishing to conduct the same research based on the data from this study will reach the same or similar conclusions. To this end, all documents and a complete record of all decisions made and ideas put forward shall be made available.

## Results

The project was funded in December 2020 and started in April 2021. By December 2022, a total of 19 participants had registered, and the first themes of the analysis were beginning to emerge. These emerging themes are informing further rounds of interviews. Recruitment is expected to be completed in February 2023, reaching thematic saturation, with results to be published in mid-2023.

## Discussion

The future results will provide a basis for establishing action guidelines based on the experiences of parents who have suffered the death of a child in the PICU. This is intended to ensure the provision of high-quality end-of-life care that helps patients to die a dignified death and parents to better manage their grief. A review of the literature found numerous studies conducted in NICUs, but only few studies involving PICUs may serve as a reference for future lines of research.

Due to the COVID-19 pandemic, the number of children admitted to the unit has been lower (with a consequent decrease in deaths). This raises the need to recruit participants retrospectively by including parents with children who have died since 2019 in the study population. Initially, a pilot study will be conducted with particularly approachable and cooperative families to decide on the questions that will make up the interview script, as well as the most appropriate time and channel for contacting families.

A potential limitation in the execution of the study is the language barrier, as the PICU at the Gregorio Marañón University Hospital is a leading cardiology unit where admitted patients (and their families) are not fluent in Spanish. Another limitation may be linked to the impact that their child's death may have on parent participation in the study.

End-of-life care is one of the main objectives of health care. Taking an in-depth look at this issue is an opportunity to improve health care delivery and the humanization of care, to help families to manage their grief and to foster a climate conducive to a dignified death. This project is intended to maintain, strengthen, and build on a particular line of research on end-of-life care in order to contribute to care excellence in the psychosocial and spiritual dimensions by focusing on effective coping, spiritual well-being, and the adaptive grieving process.

## Appendix

Multimedia Appendix 1 Experiential characteristics.

Multimedia Appendix 2 Interview guide.

Multimedia Appendix 3 Peer review report by Programa Intramural de Impulso a la I+D+i - Fundación para la Investigación Biomédica del Hospital Gregorio Marañón (FIBHGM) - Instituto de Investigación Sanitaria Gregorio Marañón (IiSGM) (Madrid, Spain).

## Abbreviations

CPR: cardiopulmonary resuscitation
FCC: family-centered care
LTE: limit therapeutic efforts
NCP: nursing care process
NICU: neonatal intensive care unit
PICU: pediatric intensive care unit

## Data Availability

The data sets used and analyzed during this study are available from the corresponding author on reasonable request.
