# Supplementary material for: Parent Experiences of Child Loss and End-of-Life Care in a Pediatric Intensive Care Unit: Protocol for a Qualitative Study
Source: JMIR Res Protoc. 2023 Mar 22;12:e43756. doi: 10.2196/43756 (PMC10131923; doi:10.2196/43756)
Supplement: Multimedia Appendix 1 [file resprot_v12i1e43756_app1.docx]

| EXPERIENTIAL CHARACTERISTICS TO BE CONSIDERED IN PURPOSIVE SAMPLING  Age of the child (infants, schoolchildren, and adolescents to ensure a representative sample) |
| --- |
| Duration of admission in the PICU (including stays that become chronic and expected deaths, as well as traumatic events leading to admission to the PICU with a sudden and unexpected death) |
| Reason for admission to the PICU (chronic, acute condition) |
| Prior re-admissions to the PICU |
| Personal history |
| Cause of death and average time between death and interview |
| Number of family deaths experienced (as these may condition their experience) |
| Family history |
| No. of family members (siblings, etc.) |
| Family support |
| Primary caregiver (adequate representation of parents will be sought to ensure richness of information) [8] |
| Family socio-economic status |
| Primary caregiver education level |
| Religion / beliefs |
