# Supplementary material for: Parent Experiences of Child Loss and End-of-Life Care in a Pediatric Intensive Care Unit: Protocol for a Qualitative Study
Source: JMIR Res Protoc. 2023 Mar 22;12:e43756. doi: 10.2196/43756 (PMC10131923; doi:10.2196/43756)
Supplement: Multimedia Appendix 2 [file resprot_v12i1e43756_app2.docx]

| **Interview Guide**   1. Could you describe the circumstances leading up to the admission of your child to the Intensive Care Unit? |
| --- |
| 1. How do you remember the moment you were informed about your child’s prognosis and the possibility that they could pass away or of a limited therapeutic effort? |
| 1. Where and in what circumstances did you receive this news? |
| 1. Who else was present in this moment? |
| 1. What aspects do you wish had been taken into consideration? |
| 1. What aspects do you consider made you aware of the reality of the situation and provided emotional support in those moments? |
| 1. To what degree do you feel you played an active role in decision making regarding care at the end of your child’s life? |
| 1. (If applicable) To what degree do you think your child was able to take part in this decision-making process? |
| 1. In which aspects of your child’s end-of-life care would you have liked to have been more involved? |
| 1. To what degree do you feel you were able to be there for your child when they passed? |
| 1. How do you remember the moment you said goodbye to your child? |
| 1. To what degree do you consider you had the time, space, and emotional support that you needed to be able to say goodbye to your child properly? |
| 1. How would you describe the support you received from the care team in the last moments of your child’s life? |
| 1. To what degree do you consider it important that you know the lead caregiver in those last moments? |
| 1. To what degree do you think your emotional and spiritual needs were met? |
| 1. What resources do you think would have helped better support you throughout this experience? |
| 1. What would you have liked to have been different? |
| 1. What aspects do you think could have been better managed by the care team? |
| 1. What advice would you give to the care team to make the experience less difficult for families going through a similar experience to yours in future? |
| 1. How did you react to the letter of condolences you received from the hospital along with your child’s medical reports? |
| 1. (If answer is negative) What would you have preferred the letter of condolences to be like? |
| 1. To what degree do you consider the contact you had with the care team after your child’s passing to have contributed, or may contribute, to coping with loss? |
| 1. What aspects would you want to discuss if you were to meet with the care team after your child’s passing? |
| 1. How do you think your experience in the ICU has affected your grieving process? |
| 1. What strategies and resources are supporting you throughout your grieving process? |
