# Supplementary material for: Parent Experiences of Child Loss and End-of-Life Care in a Pediatric Intensive Care Unit: Protocol for a Qualitative Study
Source: JMIR Res Protoc. 2023 Mar 22;12:e43756. doi: 10.2196/43756 (PMC10131923; doi:10.2196/43756)
Supplement: Multimedia Appendix 3 [file resprot_v12i1e43756_app3.pdf]

Programa Intramural de Impulso a la I+D+i del año 2020

Hoja de Evaluación

Proyectos de Investigación en Enfermería

Baremo / Candidato	MÉRITOS CURRICULARES IP (15 ptos)		MÉRITOS GRUPO (15 ptos)		PROPUESTA (70 ptos)		TOTAL
	Publicaciones JCR (10 ptos)	Otros (proyectos, patentes, GPC, EECC…) (5 ptos)	Publicaciones, Proyectos, patentes, GPC… (10 ptos)	Capacidad Formativa (5 ptos)	Calidad científico-técnica (50 ptos)	Interés estratégico (20 ptos)	
Sara Alcón Nájera (Intens. Pediát.)	2	3	8	5	43	15	**76**

JUSTIFICACION CIENTIFICA

La candidata presenta una actividad investigadora breve, pero su curriculum tiene algunos apuntes de interés, como es la recepción de un premio nacional, por un trabajo ya ligado a su área de interés en relación con el niño atendido en la Unidad de Cuidados Intensivos Pediátricos. Dos estancias de voluntariado en áreas de gran vulnerabilidad contribuyen también a complementar un perfil atractivo. El proyecto que presenta tiene un interés indudable para el hospital y el servicio donde se llevará a cabo. Relacionado con las áreas de trabajo e investigación de la IP y, por tanto, sigue la línea de la preocupación del servicio por los cuidados alrededor de la muerte en la UCIP. El proyecto está bien justificado y el diseño es apropiado a los objetivos que se plantean. Como mejoras, se recomienda revisar el guion de entrevista para modificar las preguntas dicotómicas, sustituyéndolas por otras más abiertas. Se recomienda, asimismo, que incluyan la posibilidad de grabación únicamente en audio, para garantizar una respuesta afirmativa de al menos los 12 progenitores que deben participar en el estudio. El presupuesto será escaso para lo que se plantea, pero podrán resolverlo con alguna otra financiación, si fuera posible. Este proyecto podría dar lugar a una tesis doctoral, si la IP así lo decide. En ese caso, se recomienda incorporar la perspectiva de los profesionales, para enriquecer la investigación e incorporar a todos los actores en una estrategia de mejora continua de la atención al niño que muere en la UCIP, su familia y los profesionales que les atienden.

Inscrita en el Registro de Fundaciones de la Comunidad de Madrid, Hoja Personal 285, Inscripción 1ª, Tomo XXXIIII, Folios 302 y siguientes- C.I.F. G-83195305
